# Role of radiotherapy in refractory/relapsed classical Hodgkin lymphoma in the era of targeted therapies and immunotherapy

**DOI:** 10.1016/j.ctro.2026.101221

**Published:** 2026-06-17

**Authors:** Sukeina Baouia, Rafik Nebbache, Côme Bommier, Florian Chevillon, Emmanuel Fardeau, Catherine Thieblemont, Laurent Quero

**Affiliations:** a Service Oncologie-Radiothérapie, Hôpital Saint Louis, Paris, France; b Service Hémato-Oncologie, Hôpital Saint Louis, Paris, France; c Université Paris Cité, Paris, France; d Service Hématologie Adolescents - Jeunes Adultes (AJA), Hôpital Saint Louis, Paris, France; e Unité INSERM U1160, Université Paris Cité, Paris, France

**Keywords:** Hodgkin, refractory, relapse, radiotherapy, immunotherapy

## Abstract

Relapsed or refractory classical Hodgkin lymphoma (R/R cHL) remains a complex therapeutic challenge despite major advances in systemic treatment. Salvage therapy followed by autologous stem cell transplantation (ASCT) remains the standard of care for eligible patients, but contemporary pre-transplant strategies increasingly incorporate brentuximab vedotin (BV) and, more recently, PD-1 inhibitors, which have reshaped the salvage landscape and challenged chemotherapy-only approaches. In this evolving context, radiotherapy (RT) continues to play an important role, although its indications have become more selective and individualized. Available data suggest that RT is particularly relevant for local control of residual disease, limited relapse, or metabolically active lesions, especially in the peri-transplant setting and after novel agents. Peri-transplant RT appears most useful in patients at high risk of locoregional relapse, including those with bulky disease, primary refractory lymphoma, residual PET positivity, or incomplete response before ASCT. By contrast, evidence supporting RT after or in combination with novel agents remains limited, largely derived from small, retrospective studies. Data on RT after BV are scarce but suggest excellent local control in carefully selected patients, while the combination of RT and PD-1 blockade appears especially promising, with encouraging efficacy signals from case reports, retrospective series, and recent prospective pediatric/AYA data. Modern RT techniques (IMRT/VMAT, IGRT, DIBH, and involved-site RT) enable response-adapted treatment delivery with improved normal tissue sparing. Overall, RT remains a valuable component of salvage strategies in R/R cHL in the era of BV and PD-1 inhibitors, but prospective studies are needed to better define patient selection, sequencing, target volumes, and dose prescriptions.

## Highlights

• BV and PD-1 inhibitors have reshaped salvage strategies in R/R cHL.
• The place of radiotherapy in this setting remains uncertain.
• This review summarizes available data on peri-transplant RT.
• It also discusses RT after BV and immune checkpoint inhibitors.
• Modern RT may support safer and more tailored salvage approaches.

## 1 Introduction

Classical Hodgkin lymphoma (cHL) is a highly curable hematologic malignancy, with contemporary first-line strategies achieving overall survival rates exceeding 90%, including in advanced-stage disease [1], [2], [3], [4], [5]. Recent analyses from the German Hodgkin Study Group (GHSG) further confirmed the favorable long-term outcomes achieved with modern first-line treatment approaches [6]. Nevertheless, a subset of patients experiences primary refractory disease or relapse and require salvage treatment strategies combining different therapeutic modalities [7], [8]. In this setting, salvage therapy followed by autologous hematopoietic stem cell transplantation (ASCT) remains the standard of care for eligible patients after first-line treatment failure, particularly in those at high risk of relapse, such as patients with relapse occurring within 12 months of treatment completion, disseminated relapse, or relapse within a prior radiotherapy field; pre-transplant salvage strategies may now include chemotherapy alone or regimens incorporating brentuximab vedotin (BV) and/or PD-1 inhibitors [8], [9], [10].

The emergence of PD-1 inhibitors (nivolumab, pembrolizumab) has profoundly changed the management of relapsed/refractory cHL (R/R cHL). They are now integrated into treatment algorithms, particularly after failure of ASCT-based intensification and/or after exposure to BV [11], [12].

In this context, radiotherapy (RT) continues to play a strategic role in classical Hodgkin lymphoma, with established indications in frontline combined-modality treatment for selected patients and an evolving role in the relapsed/refractory setting, particularly as consolidative or local salvage therapy after novel agents in patients with incomplete radiologic or metabolic response, focal residual disease, or oligoprogression during systemic therapy.

Advances in radiation techniques (IMRT/VMAT, IGRT, DIBH, and even proton therapy) together with reduced treatment volumes (ISRT) now make it possible to deliver RT after salvage treatment while limiting exposure to organs at risk, with the goals of improving disease control and reducing late toxicities, particularly cardiac, pulmonary, and breast toxicities.

**Fig. 1:**
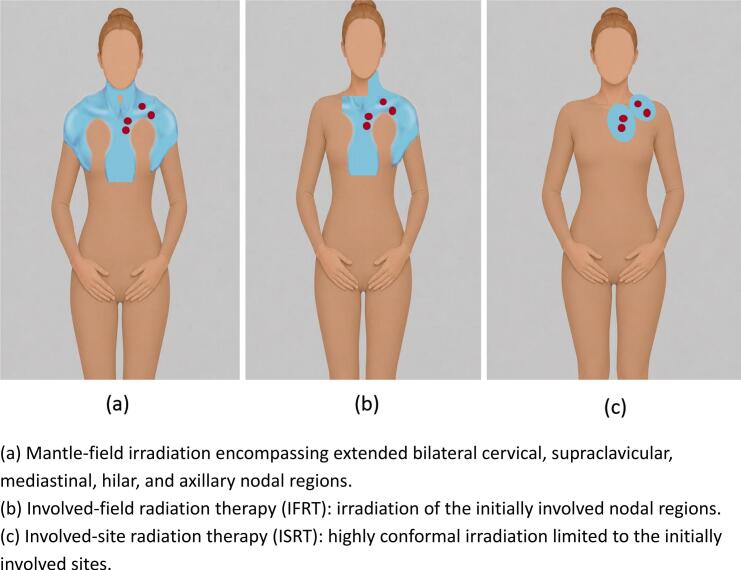
Historical evolution of radiation treatment volumes in lymphoma illustrated in the same patient: from mantle field to ISRT. Blue: field of irradiation. Red: sites of relapse. (a) Mantle-field irradiation encompassing extended bilateral cervical, supraclavicular, mediastinal, hilar, and axillary nodal regions. (b) Involved-field radiation therapy (IFRT): irradiation of the initially involved nodal regions. (c) Involved-site radiation therapy (ISRT): highly conformal irradiation limited to the initially involved sites. (For interpretation of the references to colour in this figure legend, the reader is referred to the web version of this article.)

Fig. 1 illustrates the evolution from historical extended-field irradiation to contemporary ISRT concepts and modern conformal dose delivery.

Beyond its locoregional effect, RT may also potentiate the activity of immune checkpoint inhibitors (ICIs) against distant disease through immunologic mechanisms (the abscopal effect), opening the door to combined treatment strategies.

ILROG guidelines emphasize that RT remains an important component of salvage therapy in R/R cHL and should be used thoughtfully according to disease extent, anatomic distribution, and response to systemic treatment [13]. These guidelines particularly highlight the value of RT in limited relapse, bulky disease, or critical sites, as well as in the setting of residual disease or incomplete metabolic response before or after intensification. ILROG recommends involved-site radiotherapy (ISRT) volumes, with doses generally ranging from 30 to 36 Gy depending on the clinical context, and stresses the importance of histologic confirmation of relapse or refractory disease whenever feasible, as well as PET integration when defining indications and target volumes.

In this review, we discuss the role of RT in combination with PD-1 inhibitors and BV in R/R cHL, focusing on the feasibility, efficacy, and safety of these combined approaches.

## 2 Treatment of relapsed/refractory cHL

For eligible patients, the conventional standard of care for R/R cHL consists of salvage chemotherapy followed by ASCT [8]. Historically, this approach has cured approximately 50% of patients [14], [15]. In current practice, however, second-line treatment increasingly relies on BV-containing chemotherapy regimens in transplant-eligible patients not previously exposed to BV, in order to improve metabolic response before ASCT. In the phase II BRaVE study, BV-DHAP yielded an 81% metabolic complete response rate and a 2-year PFS of 74%, supporting better disease control than that historically achieved with chemotherapy-only salvage regimens, although long-term cure rates remain to be defined [16].

Risk factors for post-transplant failure are largely related to disease chemosensitivity. Incomplete response to salvage therapy and, most importantly, a positive pre-ASCT FDG-PET scan indicating metabolically active residual disease are among the strongest predictors of poor outcome after transplantation [8], [17]. Additional adverse prognostic features include unfavorable disease kinetics, such as primary refractory disease or early relapse after first-line therapy, as well as markers of high tumor burden or aggressive disease biology at the time of salvage, including extranodal involvement, stage IV disease, bulky disease, and, in some series, B symptoms and poor performance status [18]. Together, these factors are consistently associated with a higher risk of post-ASCT progression. For patients who are not candidates for transplantation, BV remains an established treatment option, whereas PD-1 inhibitors, including nivolumab and pembrolizumab, have become a major alternative, particularly after ASCT failure, in transplant-ineligible patients, and increasingly across later-line treatment strategies [8], [11], [12].

### 2.1 Brentuximab vedotin therapy

BV is an anti-CD30 antibody-drug conjugate linked through a protease-cleavable linker to the microtubule-disrupting agent monomethyl auristatin E (MMAE), thereby targeting CD30, which is strongly overexpressed on the surface of Reed-Sternberg cells.

BV demonstrated efficacy after ASCT in the pivotal Younes et al. study, with an overall response rate of 75% and a complete response rate of 34% [19]. In the post-ASCT setting, the phase III AETHERA trial subsequently confirmed its value as consolidation therapy in high-risk patients, with a significant progression-free survival (PFS) benefit versus placebo. However, no overall survival (OS) benefit was demonstrated, because crossover was allowed and widely used (85% of patients in the placebo arm), underscoring the efficacy of BV at relapse after ASCT. At 5 years, PFS and OS were 59% versus 41% and 76% versus 77%, respectively, favoring BV for PFS without any difference in OS [20]. BV was generally well tolerated, with sensory peripheral neuropathy (56%) and neutropenia (35%) as the main reported adverse events. These findings position BV primarily as a major historical option in the post-ASCT setting, either for relapse treatment or for consolidation. In the salvage setting, the combination of BV and bendamustine has also shown substantial activity, with an overall response rate of 78% and a complete response rate of 43% in the phase 2 cohort of a multicenter phase 1–2 study, supporting its use as an effective bridging strategy before ASCT in relapsed/refractory cHL. This combination is particularly attractive when the therapeutic goal is to deepen response before transplantation, although the available evidence remains based on a non-comparative study [21].

### 2.2 PD-1 inhibitor therapy

Reed-Sternberg cells are characterized by overexpression of PD-L1 and PD-L2, driven in part by alterations at the 9p24.1 locus and activation of the JAK/STAT pathway, leading to inhibition of the T-cell immune response through PD-1 [22]. On this biologic basis, PD-1 inhibitors, particularly nivolumab and pembrolizumab, have demonstrated substantial activity in R/R cHL. In KEYNOTE-087 and CheckMate 205, conducted mainly in heavily pretreated patients, often after ASCT, overall response rates were in the range of 70%, with durable responses in a meaningful proportion of patients [11], [23].

A systematic review and meta-analysis including 15 studies and 1125 patients confirmed this activity, reporting an overall response rate of 74%, a complete response rate of 40%, 2-year overall survival of 89%, and 2-year progression-free survival of 44% [24].

Beyond their activity as single agents in later-line settings, PD-1 inhibitors are increasingly challenging BV in earlier salvage strategies before ASCT. In particular, when combined with chemotherapy backbones, they have produced very high complete response rates in transplant-eligible patients with R/R cHL. In the phase II study by Moskowitz et al., pembrolizumab plus GVD achieved an overall response rate of 100% and a complete response rate of 95%, with 95% of evaluable patients proceeding to high-dose chemotherapy followed by ASCT [25]. Likewise, in the phase II study by Mei et al., nivolumab plus ICE yielded an overall response rate of 100% and a complete response rate of 86% in a high-risk population, with 2-year PFS and OS of 88% and 100%, respectively, and a 2-year post-ASCT PFS of 94% [26]. Taken together, these results suggest that PD-1-based salvage regimens may now rival, and in some settings potentially outperform, BV-based strategies in achieving deep pre-transplant responses.

### 2.3 Comparison of PD-1 inhibitors and BV

The KEYNOTE-204 trial, which directly compared pembrolizumab with BV, showed a PFS advantage in favor of pembrolizumab [12]. These findings suggest that, in patients who relapse after ASCT or are ineligible for transplantation, PD-1 blockade is more effective than BV monotherapy, which has helped establish PD-1 inhibitors as a preferred option in this setting.

More recently, a large international multicenter retrospective study further suggested that PD-1-based salvage may also outperform BV-containing approaches before ASCT. In this analysis of 1280 patients who underwent ASCT, pre-transplant exposure to PD-1 blockade was associated with a significantly higher 2-year post-ASCT PFS than BV without PD-1 inhibitors or chemotherapy alone (88.2% vs 70.2% vs 67.4%, respectively; *P* < 0.0001). Notably, this advantage persisted among patients in complete metabolic response before ASCT, with a 2-year post-ASCT PFS of 94.4% in the PD-1 group compared with 78.5% in the BV group and 75.0% in the chemotherapy group. In multivariable analysis, PD-1-based salvage remained independently associated with improved post-ASCT PFS, whereas BV without PD-1 blockade did not differ significantly from chemotherapy alone [27]. Taken together, these data suggest that PD-1-based strategies may not only challenge BV in the post-ASCT or transplant-ineligible setting, but may also represent a more effective pre-transplant salvage platform for achieving deeper remission and improving post-ASCT outcomes.

### 2.4 PD-1 inhibitor + BV combination

Combinations of BV and PD-1 blockade, especially BV plus nivolumab, have produced high complete response rates, particularly in pre-transplant salvage strategies [10]. They therefore appear to be promising approaches for deepening response before treatment intensification [28]. This activity has also been confirmed in children, adolescents, and young adults (CAYA), a population in whom long-term toxicity reduction and treatment de-escalation remain particularly important considerations. In pediatric and AYA patients, the use of highly conformal ISRT approaches and response-adapted strategies aims to minimize late cardiovascular toxicity, endocrine sequelae, and secondary malignancies. By contrast, in adult patients with R/R cHL, RT may be used across a broader spectrum of indications, ranging from curative-intent consolidation or local salvage strategies to palliative treatment for symptom control, localized refractory disease, or bridging approaches before transplantation or cellular therapies.

In the standard-risk cohort of CheckMate 744, Harker-Murray et al. reported a complete metabolic response (CMR) rate of 59% after nivolumab plus BV induction, increasing to 94% before consolidation with response-adapted BV plus bendamustine intensification, with a 1-year PFS of 91% [29]. Similarly, in the low-risk cohort reported by Daw et al., a response-adapted nivolumab-BV-based strategy achieved a CMR rate of 93% and a 3-year PFS of 95% [7]. These pediatric and AYA data further support BV plus PD-1 blockade as a highly active salvage strategy across age groups.

### 2.5 Radiotherapy and ASCT

Available data on peri-transplant RT in R/R cHL suggest a benefit primarily in local disease control and, in some series, in progression-free survival, but they are based mainly on retrospective studies with limited sample sizes (Table 1). RT was most often delivered after ASCT, with median doses in the range of 30 to 36 Gy. In the series reported by Wilke et al., consolidative RT after ASCT was associated with a significant improvement in 2-year PFS (67% vs 42%), without a survival benefit [30]. A subgroup analysis showed a greater benefit from consolidative RT in patients with bulky disease, B symptoms, primary refractory disease, or a partial response before transplant. In addition, B symptoms and refractory disease were independent predictors of progression after ASCT. In the multicenter study by Milgrom et al., RT did not significantly improve PFS or OS in the overall cohort, but it reduced the risk of local relapse after adjustment, with a particularly clear benefit in high-risk patients, defined by primary refractory disease and/or persistent PET hypermetabolism at the time of ASCT [31]. More recent data from Levis et al. confirm that prognosis after a peri-transplant strategy is largely determined by tumor burden and the quality of response to salvage therapy: a short interval before relapse, irradiation of more than three sites, and failure to achieve a complete metabolic response were associated with poorer outcomes, whereas limited disease and complete metabolic response before or after RT were favorable factors [9]. Finally, the study by Kahn et al. suggested a greater benefit in patients with bulky disease [32].

**Table 1:** Main studies evaluating peri-transplant radiotherapy in relapsed/refractory classical Hodgkin lymphoma undergoing ASCT.

Study	Year	N (RT/total)	Setting	RT dose (Gy)	PFS / local control	OS	Grade ≥ 3 toxicity	Adverse prognostic factors / subgroups potentially benefiting from RT
Kahn et al.	2011	46/92	Pre-ASCT (83%)	30 (21–45)	∼65% vs ∼54% at 5 years	∼87% vs ∼92% at 5 years	28% vs 2%	Bulky disease (>5 cm)
Levis et al.	2016	21/73	Post-ASCT (71%)	30 (25.2–43.2)	62% vs 60% at 3 years	87% vs 80% at 3 years	0%	–
Milgrom et al.	2017	22/189	Mostly post-ASCT (95%); RT vs no RT	36 (25.2–41.4)	∼65% vs ∼58% at 2 years	∼87% vs ∼85% at 2 years	5% (1/22)	Refractory disease; persistent hypermetabolic disease on pre-ASCT PET
Wilke et al.	2017	32/80	Post-ASCT; RT vs no RT	30.6 (16–44)	67% vs 42% at 2 years	100% vs 93% at 2 years	6% (2/32)	B symptoms; refractory disease

Abbreviations: RT, radiotherapy; PFS, progression-free survival; OS, overall survival; ASCT, autologous stem cell transplantation; PET, positron emission tomography.

Post-ASCT RT appears to be generally well tolerated in the most recent series, with few severe toxicities reported. By contrast, in the Kahn et al. series, pre-ASCT RT was associated with increased severe toxicity, probably related to the concomitant use of busulfan-based conditioning.

Overall, peri-transplant RT seems most relevant in selected patients at high risk of locoregional relapse, especially those with bulky disease, refractory disease, residual PET positivity, or an incomplete response before ASCT.

### 2.6 Radiotherapy and novel agents

In the setting of rapidly evolving systemic therapy for R/R cHL, the role of radiotherapy is being redefined in a more selective way. The introduction of BV and, especially, PD-1 inhibitors, now used earlier in the treatment sequence, together with the development of PET response-adapted strategies, has led to a more individualized use of irradiation. RT may be offered for local control of limited relapses, consolidation of residual sites, irradiation of lesions that remain metabolically active after systemic therapy, or in patients with an incomplete response when optimal local control is necessary to support the next therapeutic step, particularly in preparation for ASCT or another subsequent treatment. In this setting, combining or sequencing RT with BV or PD-1 blockade is attracting increasing interest, with the goal of improving locoregional control while fitting into an overall salvage strategy.

#### 2.6.1 Radiotherapy and BV

Data specifically addressing the RT-BV combination in R/R cHL remain extremely limited (Table 2). The main published series is that reported by Zhao et al., who evaluated 19 patients treated with RT after failure of BV and/or checkpoint inhibitors. In this series, radiotherapy delivered to hypermetabolic residual lesions using modern techniques (IMRT/VMAT) achieved a complete metabolic response in all evaluable patients, with a 1-year PFS of 84.4%, 100% local control, and no grade ≥ 3 toxicity [33]. The relapses that occurred were mainly outside the irradiated volume, suggesting that RT primarily contributed excellent local control in this setting, whereas the main risk remained systemic. These findings support a personalized use of RT after BV, particularly in patients with localized residual disease and a need for optimal local control. They should, however, be interpreted cautiously given the small sample size, the heterogeneity of prior treatments, and the inclusion of patients previously exposed to ICIs. Dozzo et al. also reported a case of R/R cHL treated with BV delivered concurrently with mantle-field RT (36 Gy), with good tolerance and a response that made subsequent haploidentical allogeneic transplantation possible [34]. The patient was in complete response 5 months after transplantation.

**Table 2:** Main study evaluating radiotherapy after brentuximab vedotin and/or immune checkpoint inhibitors in relapsed/refractory classical Hodgkin lymphoma.

Study	Year	N	Setting	RT dose	Response	Outcome	Grade ≥ 3 toxicity
Zhao et al.	2024	19	RT after failure of BV and/or ICIs	Median CTV dose: 26.4 Gy; median GTV boost: 36 Gy	ORR 100%	1-year PFS 84.4%; local control 100%; 1-year OS 100%	0

Abbreviations: RT, radiotherapy; BV, brentuximab vedotin; ICI, immune checkpoint inhibitor; ORR, overall response rate; PFS, progression-free survival; OS, overall survival; CTV, clinical target volume; GTV, gross tumor volume.

#### 2.6.2 Radiotherapy and PD-1 inhibitors

Beyond local tumor cytoreduction, radiotherapy (RT) may exert clinically meaningful immunomodulatory effects in the era of immune checkpoint inhibitors. Preclinical and translational studies have shown that RT can enhance tumor immunogenicity through the induction of immunogenic cell death, release of tumor-associated antigens and damage-associated molecular patterns (DAMPs), and activation of type I interferon and cGAS/STING-related pathways. These mechanisms may promote dendritic cell maturation, antigen processing and presentation, as well as recruitment and priming of cytotoxic T cells within the tumor microenvironment. RT has also been associated with modulation of PD-L1 expression and increased immune cell infiltration, providing a biological rationale for combinations with PD-1 blockade [35], [36], [37]. This may be particularly important in classical Hodgkin lymphoma, a disease characterized by profound immune microenvironment interactions and frequent alterations in MHC class I expression [38]. Under selected conditions, these immune effects may extend beyond the irradiated field and contribute to systemic antitumor responses, referred to as the abscopal effect. Although the clinical significance of these mechanisms in R/R cHL remains incompletely defined, they provide a strong biological rationale for integrating modern involved-site radiotherapy with immune-based salvage strategies.

However, the optimal RT dose, fractionation, and sequencing relative to ICI administration to maximize immune synergy remain undefined.

Available data on the combination of PD-1 blockade and radiotherapy in R/R cHL remain limited, but they suggest clinically meaningful activity, especially in heavily pretreated patients who have failed ASCT and BV (Table 3). The initial evidence comes from case reports and small series, suggesting not only excellent local control but also, in some cases, a possible systemic abscopal effect. Qin et al. reported three durable complete responses, both local and distant, after nivolumab combined with RT given in different sequences [39]. Likewise, Quéro et al. observed a complete metabolic response in all four heavily pretreated patients treated with anti-PD-1 therapy followed by involved-site radiotherapy after a median follow-up of 13 months, although all four patients developed grade 1–2 pulmonary toxicity [40]. De Forceville et al. also reported two cases of complete response after radiotherapy combined with nivolumab in patients who relapsed after ASCT and BV consolidation [41].

**Table 3:** Main studies evaluating radiotherapy and PD-1 inhibitors in relapsed/refractory classical Hodgkin lymphoma.

Study	Year	N	Setting	RT dose	Best response	Outcome	Grade ≥ 3 toxicity
Michot et al.	2016	1	Post-ASCT, progression on anti-PD-1	30 Gy	CR	2-month PFS/OS: 100%	0
Wight et al.	2018	1	Post-ASCT, progression on anti-PD-1	20 Gy, then 24–36 Gy	CR	2-month PFS/OS: 100%	NR
Qin et al.	2018	3	Pre−/post-ASCT, sequential RT + nivolumab	20 Gy; 36–40 Gy	CR 3/3	2-year PFS/OS: 100%	NR
De Forceville et al.	2019	2	Post-ASCT, RT before nivolumab	30–36 Gy	CR 2/2	1-year PFS/OS: 100%	0
Quéro et al.	2019	4	Mostly post-ASCT, RT after anti-PD-1	22–36 Gy	CR 4/4	1-year PFS/OS: 100%	0*
Lucchini et al.	2021	12	RT before, during, or after anti-PD-1-based therapy	Median 30 Gy (30–40)	ORR 100%; CR 58% after ICI-RT; 11/12 in CR at 18 months	18-month PFS 92%; 11/12 alive	2/12
CheckMate# 744	2025	28	Low-risk CAYA patients; nivolumab + BV followed by ISRT	30.0–30.6 Gy	CR 23/28	3-year PFS 95%; OS 100%	7/28

Abbreviations: RT, radiotherapy; CR, complete response; ORR, overall response rate; PFS, progression-free survival; OS, overall survival; ASCT, autologous stem cell transplantation; ISRT, involved-site radiotherapy; CAYA, children, adolescents, and young adults; NR, not reported. Notes: * No grade ≥ 3 toxicity reported, although grade 1–2 pulmonary toxicity occurred in all four patients. # CheckMate 744 was conducted in a distinct clinical setting (transplant-free strategy in low-risk CAYA patients) and is therefore not directly comparable with adult post-ASCT salvage series).

The Italian multicenter retrospective series by Lucchini et al., the largest published series to date, included 12 patients treated with RT delivered concurrently with, before, or after PD-1 blockade. The overall response rate was 100% and the complete response rate after ICI-RT was 58%, with 11 of 12 patients (92%) in complete response at a median follow-up of 18 months, most often after additional consolidation with transplantation [42]. Tolerance was overall acceptable, but two patients developed grade 3 interstitial pneumonitis, warranting particular caution, especially with mediastinal irradiation.

More recently, prospective pediatric and AYA data have expanded this concept beyond the post-ASCT setting. In the low-risk cohort of CheckMate 744, Daw et al. evaluated a transplant-free, response-adapted strategy in patients aged 5 to 30 years with localized, nonirradiated relapse occurring more than 12 months after frontline therapy. Patients received nivolumab plus BV, followed by BV plus bendamustine in case of suboptimal response, and consolidative ISRT at 30.0 to 30.6 Gy. Among 28 treated patients, the complete metabolic response rate at any time before ISRT was 93%, and the 3-year progression-free survival was 95%, with no patient undergoing autologous transplantation. These results are of particular interest because they suggest that, in carefully selected low-risk patients, a PD-1 inhibitor-based strategy combined with modern ISRT may provide durable disease control while avoiding HDCT/ASCT [7].

Several prospective and retrospective studies are ongoing to evaluate the benefit of combining PD-1 inhibitors with radiotherapy in patients with R/R cHL. A North American prospective single-arm phase II study (NCT03179917) is evaluating a sequential strategy of four cycles of pembrolizumab followed by involved-site radiotherapy, with a dose of 20 Gy in patients who achieve a complete metabolic response and 36 to 40 Gy in those with histologically documented residual disease; the primary endpoint is 2-year progression-free survival. The retrospective Italian observational study ICI-RT-1 (NCT04419441) is evaluating the efficacy and safety of combining radiotherapy with ICIs (nivolumab or pembrolizumab) in R/R cHL; its primary endpoint is complete metabolic response assessed early after completion of systemic therapy or radiotherapy (2 to 3 months). Finally, the U.S. phase II RadVax/UPCC 04418 trial (NCT03495713) is exploring the addition of low-dose irradiation, 2 × 4 Gy, to sites that have not achieved a complete metabolic response after nivolumab, with complete response at 25 months as the primary endpoint.

Beyond the initial response to PD-1 inhibitors, the pattern of progression is particularly important when defining the role of radiotherapy. In the series by Burlile et al., which included 69 patients treated with ICIs, 41 patients progressed during follow-up, and more than half of these progressions (22/41; 54%) occurred exclusively at sites already present before ICI initiation [43]. Among patients with limited disease before ICI, defined as ≤5 sites on baseline PET, the cumulative incidence of progression potentially amenable to local therapy was 34%, suggesting that a meaningful subgroup might benefit from consolidative RT during or after immunotherapy. By contrast, higher tumor burden before ICI appeared to be a risk factor for progression: patients with more than five involved sites had a significantly higher risk of progression, and each additional site beyond five further increased that risk. These findings strengthen the hypothesis that a more selective RT approach may be particularly relevant for patients with initially limited disease who progress at preexisting sites, whereas more disseminated disease patterns are more likely to require systemic intensification.

Interpretation of FDG-PET/CT after PD-1 blockade may be challenging, as persistent FDG avidity does not necessarily reflect viable lymphoma and may also result from immune-related inflammatory changes, pseudoprogression, flare reactions, or other atypical response patterns described with checkpoint inhibitors. In this context, PET findings should be interpreted cautiously, ideally in conjunction with clinical evolution, repeat imaging, LYRIC (Lymphoma Response to Immunomodulatory Therapy Criteria), and histopathological confirmation whenever feasible [44], [45].

## 3 Conclusion

While the role of radiotherapy combined with chemotherapy is well established in first-line classical Hodgkin lymphoma, particularly for disease control in selected early-stage and PET-adapted strategies, indications in the relapsed/refractory setting are more heterogeneous and increasingly individualized. In the era of BV, and especially PD-1 inhibitors, radiotherapy continues to play an important role in the management of R/R classical Hodgkin lymphoma, although its indications are now increasingly selective and individualized. RT appears particularly relevant for local control of residual disease, limited relapses, or metabolically active sites. In current practice, it is often most appropriately delivered after BV or PD-1 blockade, particularly in patients with localized residual disease, whereas in the peri-transplant setting, the choice between pre- and post-ASCT radiotherapy should be tailored to disease distribution, metabolic response to salvage therapy, and the expected balance between cytoreduction and toxicity. Modern techniques, including IMRT, IGRT, DIBH for mediastinal disease, and contemporary involved-site approaches, have made RT more precise and potentially safer, allowing improved conformality, motion management, and normal tissue sparing. However, the evidence supporting RT after novel agents, especially after PD-1 inhibitors, remains limited and largely non-randomized. Among combined strategies, RT plus PD-1 blockade appears particularly promising, but available data are still based mainly on case reports, small retrospective series, and highly selected populations, while pulmonary toxicity warrants careful attention. To date, no randomized prospective data currently define the optimal integration of RT with PD-1 blockade. Further prospective studies are needed to better define optimal sequencing, indications, target volumes, and dose prescriptions according to patient and disease characteristics.

## CRediT authorship contribution statement

**Sukeina Baouia:** Writing – original draft, Writing – review & editing. **Rafik Nebbache:** Writing – review & editing. **Côme Bommier:** Writing – review & editing. **Florian Chevillon:** Writing – review & editing. **Emmanuel Fardeau:** Writing – review & editing. **Catherine Thieblemont:** Writing – review & editing. **Laurent Quero:** Conceptualization, Supervision, Project administration, Writing – original draft, Writing – review & editing.

## Declaration of competing interest

CB and have received honoraria for advisory board participation from Takeda. LQ has received financial support from MSD for a departmental project. SB, RN, EF and CT declare that they have no competing interests.
